# Hybrid of *Metapenaeus dobsoni* lectin and platinum nanoparticles exert antimicrobial and immunostimulatory effects to reduce bacterial bioburden in infected Nile tilapia

**DOI:** 10.1038/s41598-022-26719-5

**Published:** 2023-01-11

**Authors:** Sreeja Lakshmi, Abdul Salam Rubeena, Siva Bala Subramaniyan, Thiagarajan Raman, Baskaralingam Vaseeharan, Jesu Arockiaraj, Sivashanmugam Karthikeyan, Veerappan Anbazhagan, Elumalai Preetham

**Affiliations:** 1 School of Ocean Science and Technology, Kerala University of Fisheries and Ocean Studies, Panangad, Kerala India; 2 Department of Biosciences, MES College Marampally, Ernakulam, Kerala 683105 India; 3 School of Chemical and Biotechnology, SASTRA Deemed University, Thanjavur, Tamil Nadu 613401 India; 4 Department of Zoology, Ramakrishna Mission Vivekananda College, Chennai, Tamil Nadu 600004 India; 5 Nanobiosciences and Nanopharmacology Division, Biomaterials and Biotechnology in Animal Health Lab, Department of Animal Health and Management, Science Campus 6th Floor, Alagappa University, Karaikudi, India; 6 Department of Biotechnology, College of Science and Humanities, SRM Institute of Science and Technology, Kattankulathur, Chennai, Tamil Nadu 603203 India; 7 School of Bioscience and Technology, VIT University, Vellore, Tamil Nadu 632014 India; 8 Department of Marine Biology, Microbiology and Biochemistry, School of Marine Sciences, Cochin University of Science and Technology (CUSAT), Lakeside Campus Fine Arts Avenue, Cochin, India

**Keywords:** Biochemistry, Immunology, Molecular biology

## Abstract

A novel antibacterial immunostimulant using Platinum nanoparticles (PtNPs) and lectin from *Metapenaeus dobsoni* (Md-Lec) was developed. The Md-Lec and PtNPs (Pt-lec) hybrid formed through non-covalent interaction exhibits antimicrobial activity against fish specific pathogens by affecting membrane integrity and producing excess reactive oxygen species. The therapeutic efficacy of Pt-lec was demonstrated through rescuing *Aeromonas hydrophila* infected Nile Tilapia. Pt-lec prevents the infection spreading and reduces the bacterial bioburden in less than 12 h, and as a result of this the fish were restored to normalcy. To assess immunostimulation, we studied the expression of three different immune related genes, namely LEC, Myd88 and COX-2 in the gills, liver, spleen and kidney of fish under various experimental conditions. Our results showed that Pt-lec treatment appeared to be better when compared to lectin alone in enhancing the expression of Myd88 and COX-2, but LEC was not as expected. These results suggest that Pt-lec has the ability to protect Nile Tilapia against bacterial infection by restricting bacterial bioburden through their direct effects on the bacterial membrane and indirectly through their effects on host immune-related gene expression. This hybrid could have potential “green” application in fish farming in rescuing infected animals when compared to widely and unregulated antibiotics.

## Introduction

Antibiotics play a very important role in fish farms, as they help in preventing disease outbreak and also facilitate optimal growth of fishes^1^. Nevertheless unregulated use of antibiotics has lead to the development of microbial resistance^2^ and thereby caused frequent outbreaks of diseases, both bacterial and viral, in fish farms. Apart from the emergence of antibiotic resistant pathogens, overuse of antibiotics has also proved to be deleterious to other organisms, including humans that are part of a complex food web^3^. Under these circumstances alternate strategies to combat pathogens in fish farms is gaining importance and immunostimulant is one such important aspect. Immunostimulants can be easily applied and so far they are well recognized in producing changes in host immune responses. But in addition to host-effects, if these immunostimulants can prove to be equally effective on the pathogen then their application becomes much more effective. In fact immunostimulats are a good option since they do not work directly and more importantly specifically against one particular target in bacteria and hence reduce the chance of resistance development^4^.

Metal-based nanoparticles have emerged as major antibacterial agents^5,6^ and their mechanism of action includes reactive oxygen species, bacterial membrane disruption and cytotoxic killing. However, much remains unknown about their exact mechanism of action against bacteria^7^. In addition, it is also unclear if these metal nanoparticles can induce resistance development, which remains unlikely at the most. One major issue with these metal nanoparticles is their lack of specificity and hence surface functionalization of these nanoparticles could aid in better and quicker bacterial recognition and binding, thereby rapidly increasing immune activation. In addition, this increase in specificity of nanoparticles due to surface functionalization could help in reducing the actual dosage in host, and thus minimizing toxicity, if any. Lectins are carbohydrate binding proteins^8^ and have a wide variety of physiological functions including stimulating immune responses in animals through non-self recognition ^9^. Lectins, owing to their role as “pattern recognition molecules” willbe better positioned to recognise bacterial cell surface carbohydrates (= pathogen associated molecular patterns; PAMPs) and activate innate immune mechanisms by binding to their receptors on immune cells. If these lectins are conjugated with metal nanoparticles, then it will facilitate quicker recognition and killing of bacterial pathogens, in addition to stimulating host immune response.

The haemolymph of the Kadal shrimp, *Metapenaeus dobsoni* contains a 68 kDa lectin (Md-Lec), which has specificity towards mannose^10^. In this study, we have developed a non-covalent hybrid (Pt-lec) of platinum nanoparticles and Md-Lec. The hybrid exhibit excellent antimicrobial activity against fish specific pathogens (*Aeromonas hydrophila*, *Enterococcus fecalis* and *Streptococcus iniae*). Mechanistic study revealed the Pt-lec is more effective in disturbing the membrane integrity than PtNPs and also Pt-lec induce the production of excess oxidative by-product, which eventually lead to cell death. The therapeutic potential of Pt-lec was demonstrated through rescuing the infected Nile Tilapia. The result showed that Pt-lec reduces the bacterial bioburden and also induce immunostimulatory activity to overcome the bacterial infection.

## Results and discussion

### Md-Lec and platinum nanoparticles binding study

**Figure 1:**
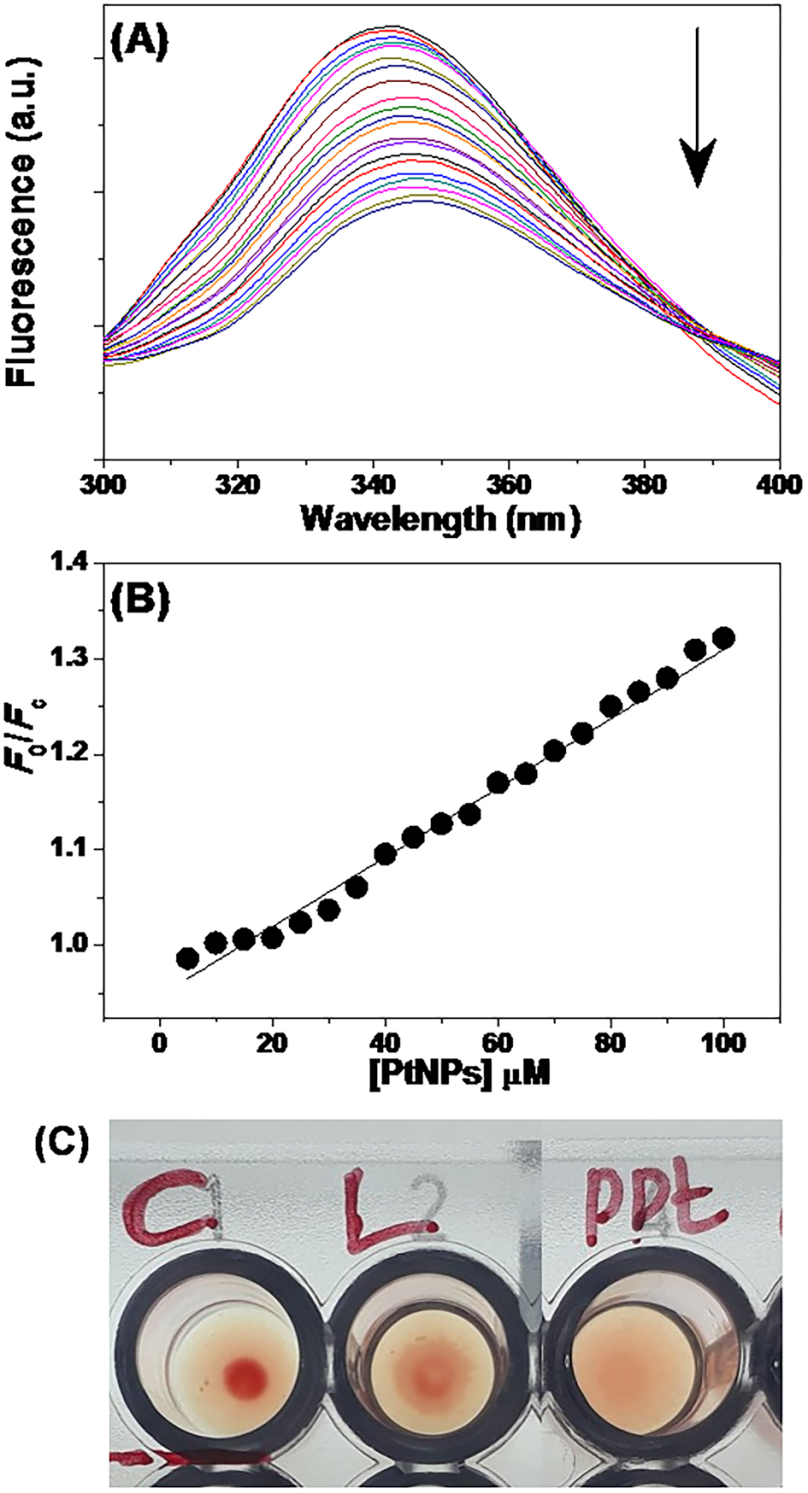
The fluorescence quenching study. (**A**) Fluorescence titration. The upper spectrum corresponds to the emission of Md-Lec alone and the remaining spectra depicts the decreasing fluorescence emission spectra with increase in the concentration of the PtNPs (0–100 µM) (**B**) Stern–Volmer plot. (**C**) Haemagglutination assay. *C* Control RBC, *L* Md-lec, *PPt* Pt-lec.

Platinum nanoparticles (PtNPs) were synthesized through reducing hexochloroplatinic acid in the presence of capping agent, Pectin^7^. The nanodimension of the PtNPs was confirmed by transmission electron microscopy (see supporting Fig. S1). The antimicrobial hybrid was prepared by incubating PtNPs and Md-Lec, isolated from the haemolymph of the Kadal shrimp, *Metapenaeus dobsoni*, for 1 h at 4 °C. The capability of Md-Lec binding non-covalently to PtNPs was judged by fluorescence quenching method^11^. The intrinsic fluorescene of Md-Lec was affected by the binding of PtNPs (Fig. 1A). It is noted from Fig. 1A, the fluorescence emission maxima of Md-Lec observed at 342 nm was quenched with the addition of PtNPs. The mechanism of quenching was analyzed by Stern–Volmer plot (Fig. 1B).

1 $$F_{0} /F_{{\text{c}}} = { 1 } + K_{{{\text{sv}}}} \left[ {{\text{PtNPs}}} \right] \, = { 1 } + K_{{\text{q}}} \tau_{0} \left[ {{\text{PtNPs}}} \right],$$

2 $$K_{{\text{q}}} = K_{{{\text{sv}}}} /\tau_{0} ,$$

where *F*_0_ is the fluorescence intensities of Md-*Lec* at 342 nm and *F*_c_ is the fluorescence intensities of Md-*Lec* in the presence of PtNPs, *K*_sv_, the Stern–Volmer fluorescence quenching constant, *K*_q_ is the biomolecular quenching constant; and τ_0_ is the average fluorescence lifetime of lectin (10^−9^ s). From the linear Stern–Volmer plot with *K*_sv_ value of 3.62 (± 0.09) × 10^3^ M^−1^ was calculated for the binding of PtNPs and Md-Lec. The obtained *K*_sv_ was comparable to reported lectin-nanoparticles interaction^11^. Generally, for any diffusion controlled quenching process the maximum *K*_q_ value is 10^10^ L mol^−1^. Interestingly, the *K*_q_ derived from *K*_sv_ was in the order of 10^12^ L mol^−1^, which is higher than the diffusion controlled process. This suggests that the mechanism of quenching is static and a non-fluorescent complex is likely formed between Md-*Lec* and PtNPs. The sugar binding site of lectin is crucial to recognize bacterial cell surface glycans, thus, the availability of the sugar binding site in Md-Lec-PtNPs (Pt-lec) hybrid was evaluated. Being a lectin, Md-Lec agglutinates human red blood cells^12^. The haemagglutination results shown in Fig. 1C reveal that Pt-lec produces clump or aggregation of erythrocytes, which appears as fluffy cotton at the bottom. The result indicates that Pt-lec retain the sugar binding site even after the formation of noncovalent hybrid. Noteworthy, the RBC treated with PBS buffer settle at the well bottom without haemagglutination (Fig. 1C).

### In vitro antimicrobial activity of the Pt-lec hybrid

**Figure 2:**
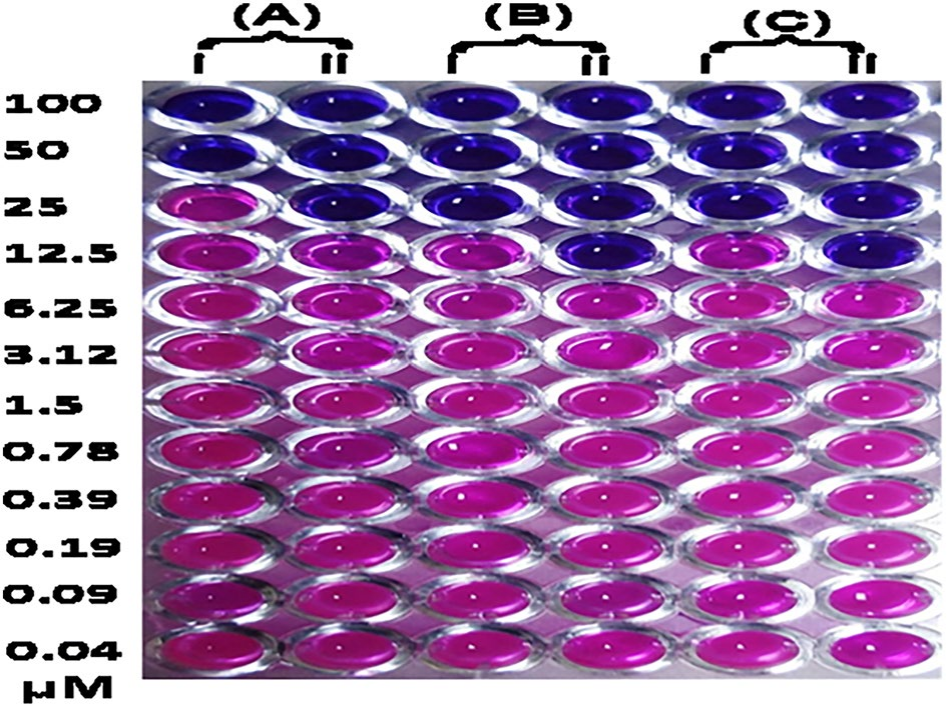
Determination of minimum inhibitory concentration of PtNPs. (**A**) *A. hydrophila*, (**B**) *E. fecalis*, (**C**) *S. iniae*. While (i) is having PtNPs alone and (ii) is having 25 µM Md-Lec and PtNPs.

The minimum concentration (MIC) of Pt-lec required to exert antimicrobial activity was determined using resazurin microtiter assay (REMA). Having confirmed the interaction between lectin and PtNPs, Pt-lec hybrid was prepared by incubating 25 µM Md-Lec and desired concentration of PtNPs at 4 °C for 2 h. The hybrid was tested against common bacterial pathogens (*A. hydrophila*, *E. fecalis* and *S. iniae*) involved in aquatic species infection. In REMA, viable cells turn the resazurin to resarufin and appear in pink color, whereas non-respiratory cells are unable to convert resazurin and appear in blue color. The REMA for PtNPs and Pt-lec hybrid is shown in Fig. 2. The MIC determined for PtNPs against *A. hydrophila*, *E. fecalis* and *S. iniae* was 50 µM, 25 µM, and 25 µM, respectively. Strikingly, the MIC of Pt-lec hybrid was two-fold lower than PtNPs, and the MIC was 25 µM, 12.5 µM, and 12.5 µM, respectively, against *A. hydrophila*, *E. fecalis* and *S. iniae*.

**Figure 3:**
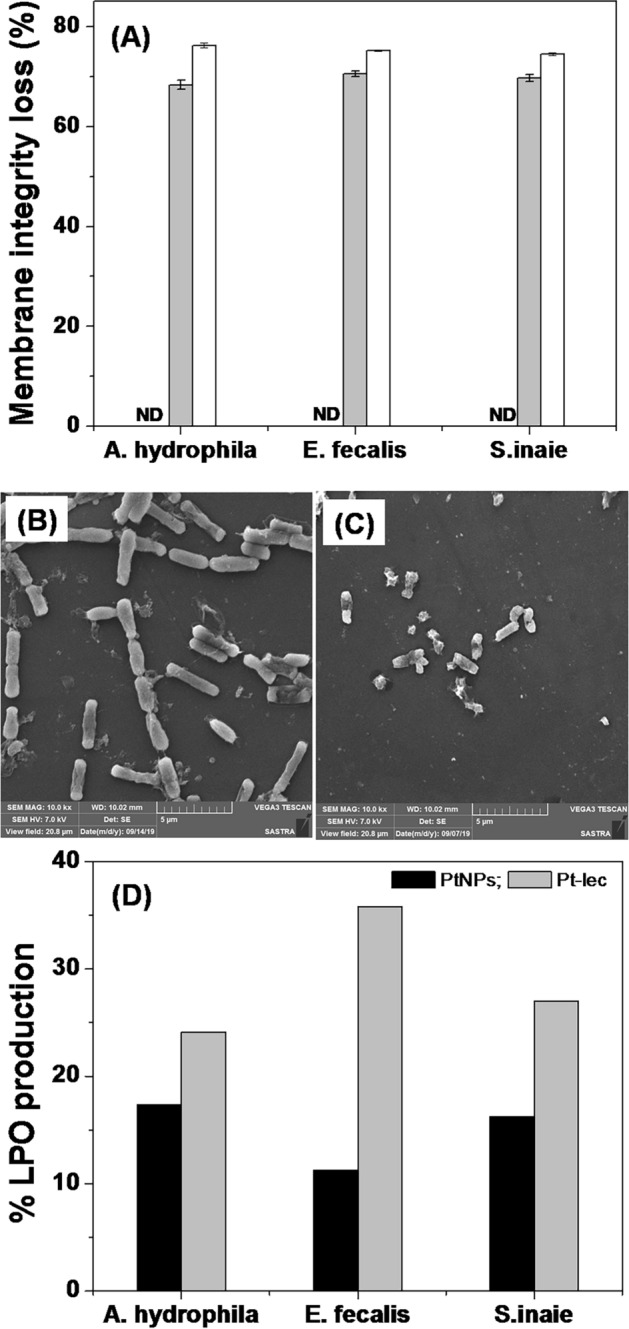
Antibacterial mechanism. (**A**) Membrane integrity assay. Bacteria were treated with 1× MIC Pt-lec (black bar) and 25 µM Triton X 100, positive control (white bar). *ND* not detectable fluorescence from untreated cells, negative control; (**B**) Representative SEM image of untreated *E. fecalis*, (**C**) *E. fecalis* treated with 1X MIC Pt-lec; (**C**) TBARS assay. Excess LPO was produced in the cells treated with 1X MIC Pt-lec.

The mechanism of action of Pt-lec hybrid was analyzed by fluorescence probe uptake assay. Generally, the cells with compromised membranes will uptake non-polar fluorescence probes and show strong fluorescence^13,14^. It is noted the aquatic pathogens exposed to 1X MIC Pt-lec hybrid engulf the fluorescence probes as evidenced by the strong fluorescence (Fig. 3A). At the same, the untreated bacteria exposed to fluorescence probe shows poor fluorescence response. The bacterial cells treated with Triton X 100, a membrane damaging agent showed strong fluorescence. These results suggest that the bacterial outer membrane integrity was affected by Pt-lec hybrid. The loss of membrane integrity on treatment with hybrid was further analyzed by scanning electron microscopy. A representative SEM image of untreated *E. fecalis* and the bacteria treated with 1× Pt-lec hybrid was shown in Fig. 3B,C. It is noted from Fig. 3B that the untreated *E. fecalis* appears in regular morphology showing rod shape with intact membranes. The cells treated with the hybrid lost the morphology and appears shrunken with damaged membranes (Fig. 3C), which corroborates with the fluorescence uptake assay. The potential of the hybrid to cause oxidative stress through increasing intracellular reactive oxygen species (ROS) generation was evaluated by measuring lipid peroxidation. The unsaturated lipids and fatty acids of bacterial membranes are vulnerable to ROS attack and forms lipid peroxide end product, malondialdehyde (MDA). An increase in ROS causes overproduction of MDA, which was measured by thiobarbituric acid reactive substances (TBARS) assay (Fig. 3D). It is noted from Fig. 3D that the 1X Pt-lec produce higher LPO than 1X PtNPs, suggesting that hybrid has superior activity due to the bacteria cell surface glycan recognition ability of Md-Lec^15,16^. The observed difference in the LPO percentage in the tested bacterium is attributed to the difference in bacterial membrane composition. Nevertheless, the result proved that the hybrid effectively affects the bacterial membrane integrity and production of LPS supports the ROS mediated destruction of bacteria^17^.

### Therapeutic efficacy of Pt-lec hybrid against infected Nile Tilapia

**Figure 4:**
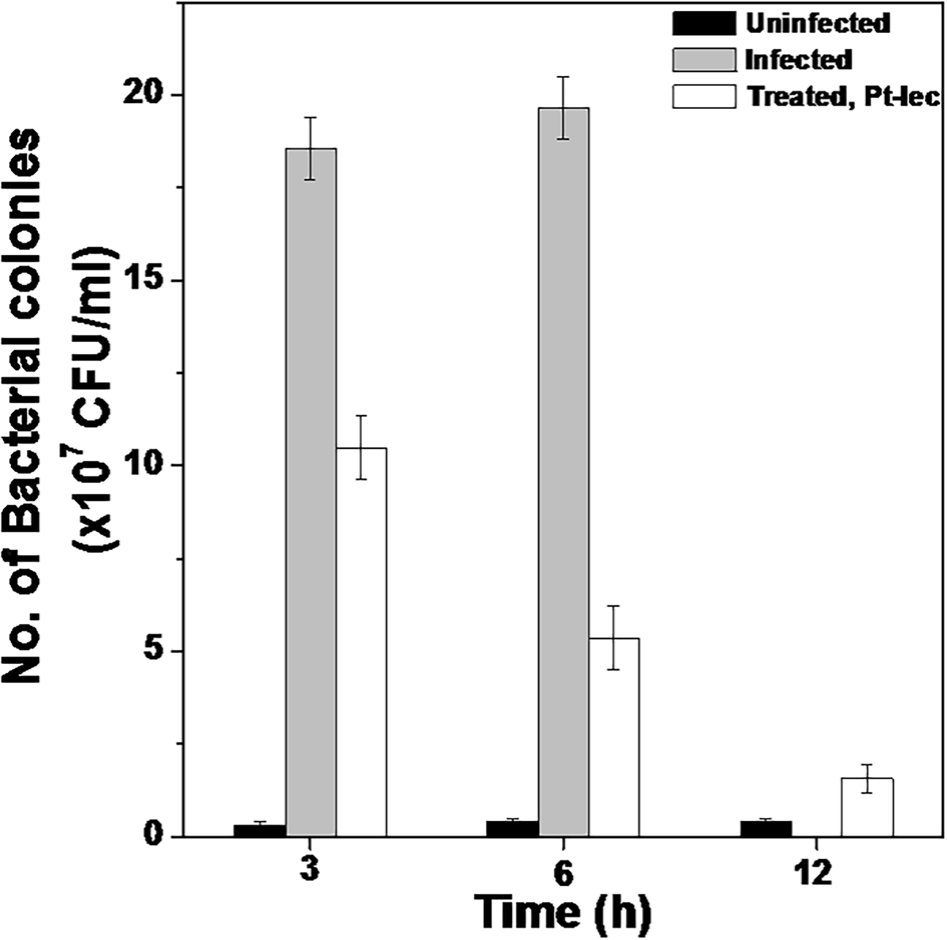
In vivo assay. The fishes treated with 1 × MIC Pt-lec showed declining number of viable cells with increasing time. At 12th h, infected fishes are not alive.

Nile tilapia (*Oreochromis niloticus*) is one of the most farmed food fishes, which is known for adaptations to hard ecological conditions. The prevalence of opportunistic bacterial contamination in aquatic system causes bacterial infectious diseases in Nile tilapia and affects the farming industry. The results from the in vitro studies suggest that Pt-lec hybrid has promising antibacterial activity against fish specific pathogens^18,19^. The competence of Pt-lec hybrid in treating in vivo bacterial infection was evaluated in Nile tilapia. Healthy fish was inoculated intramuscularly with *A. hydrophila* and the infection was allowed to be established for the first 3 h. After 3 h, the fish were divided into two groups: Group A was injected with PBS and Group B was treated with Pt-lec hybrid. Uninfected fish served as a control. Nile tilapia is known for adaptations in tougher conditions, but intramuscularly infected Group A fish succumbed to infection in 10–12 h. Strikingly, Group B fish treated with hybrid recovered from the infection and restored to normalcy in less than 12 h. In order to understand the extent of infection, the bacterial load in the muscle was analyzed by colony count assay. The muscle was dissected, digested in buffer, and plated on LB-agar. The number of bacterial colonies found in the muscle at defined time point is shown in Fig. 4. It was noted that the number of bacterial colonies in Group B fish treated with hybrid showed significant decrease after 3 h treatment, when compared to Group A fishes. After 6 h treatment the number of bacterial colonies did not decrease in Group A, but Group B showed very significant lower bacterial load, suggesting the potential of the hybrid in facilitating faster bacterial clearance. Over the time of 12 h, the bacteria were mostly eliminated in Group B, whereas Group A fishes were unable to recover from the infection and all of them died between 10 and 12 h. The result clearly showed that Pt-lec is very efficient in rescuing bacteria infected Nile tilapia and the fishes were normal within 24 h.

### Pt-lec hybrid as immunostimulant

Our experiments with antibacterial activities in vivo, showed clear reduction in bacterial load in infected fish that were treated with Pt-lec. In addition, bacteria infected and Pt-lec treated fish were also rescued from active infection that resulted in survival of the fishes. This suggests to us that apart from the direct effect of the hybrid against bacteria, these particles could also have an effect on the immune response of the fish, thereby facilitating faster clearance and better survival. To assess immunostimulation, we studied the expression of three different immune related genes, namely LEC, Myd88 and COX-2 in the gills, liver, spleen and kidney of fish under various experimental conditions (see supporting Fig. S2).

**Figure 5:**
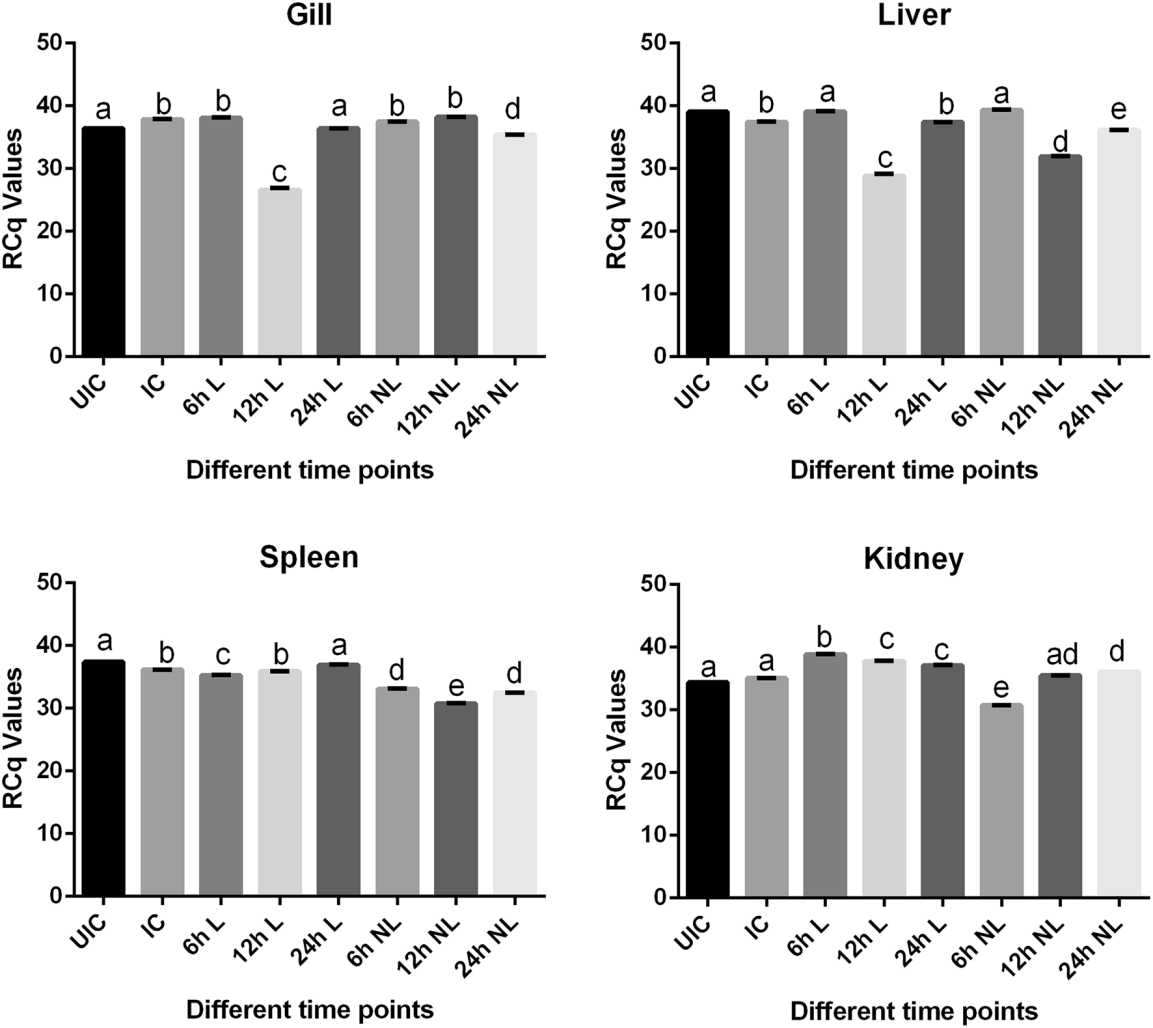
Gene expression analysis of lectin in the gills, liver, kidney and spleen of infected fish with *A. hydrophila* and treated with lectin and Pt-lec at different time intervals (6 h, 12 h and 24 h). *UIC* Uninfected control, *IC* infected control, *L* lectin treated, *NL* lectin-PtNPs hybrid treated.

The expression of these genes between lectin alone treated (lec) and Md-Lec-PtNPs (Pt-lec) treated fish after infection was compared (Fig. 5). As the results show, expression of LEC genes were found to be slightly reduced in liver (− 1.04-fold change) and spleen (− 1.03-fold change) in the case of infected control (IC), when compared to respective uninfected control (UIC). In the case of gills (1.03-fold change) and kidney (1.01-fold change) from IC group, there was a slight increase in expression when compared to the respective UIC. When compared to IC and UIC groups, LEC gene expression showed differential expression under different treatment conditions, wherein increase in expression was observed in the case of gills (6 h, 1.00-fold) and Pt-lec (12 h, 1.00-fold) and in the case of kidney most treatment conditions showed increase, all when compared to IC and NIC. In the case of liver and spleen, LEC gene expression in both lec and Pt-lec treated fish was found to be lower when compared to the respective IC and NIC. LEC gene is critical for lectin function, which forms an important innate immune component in living systems. Lectin is recognized to play an important role as a recognition molecule between pathogen and immune components of the host. By nature of their interaction with specific carbohydrates, they could be considered to be equivalent to the interaction between pattern recognition receptors and pathogen associated molecular patterns^20^. In fact, lectin has been shown to function as a critical immune molecule in terms of its role as an agglutinin^11^, opsonin^6^ and in complement activation^21^. Irrespective of the tissue analysed, LEC gene expression was observed to be slightly affected upon infection and both L and Pt-lec were able to produce slight stimulation over and above that of IC and UIC, as observed with gills and kidney. This induction in LEC gene could probably facilitate activation of both innate and adaptive immune responses in the fish and this remain to be investigated.

Myeloid differentiation factor 88 (Myd 88) is an adaptor protein required for cytokine signaling, e.g. IL-1^22^ and is also critical for much of Toll-like receptor (TLR) family signaling^22^. Bacteria detecting TLRs primarily signal through Myd 88 and thus, except for TLR3^23^, rest of the TLR members use Myd 88 for downstream signaling leading to the activation and nuclear translocation of NF-κB and MAPK^24^. Thus, Myd 88 is important for producing immune activation in terms of a proinflammatory environment.

**Figure 6:**
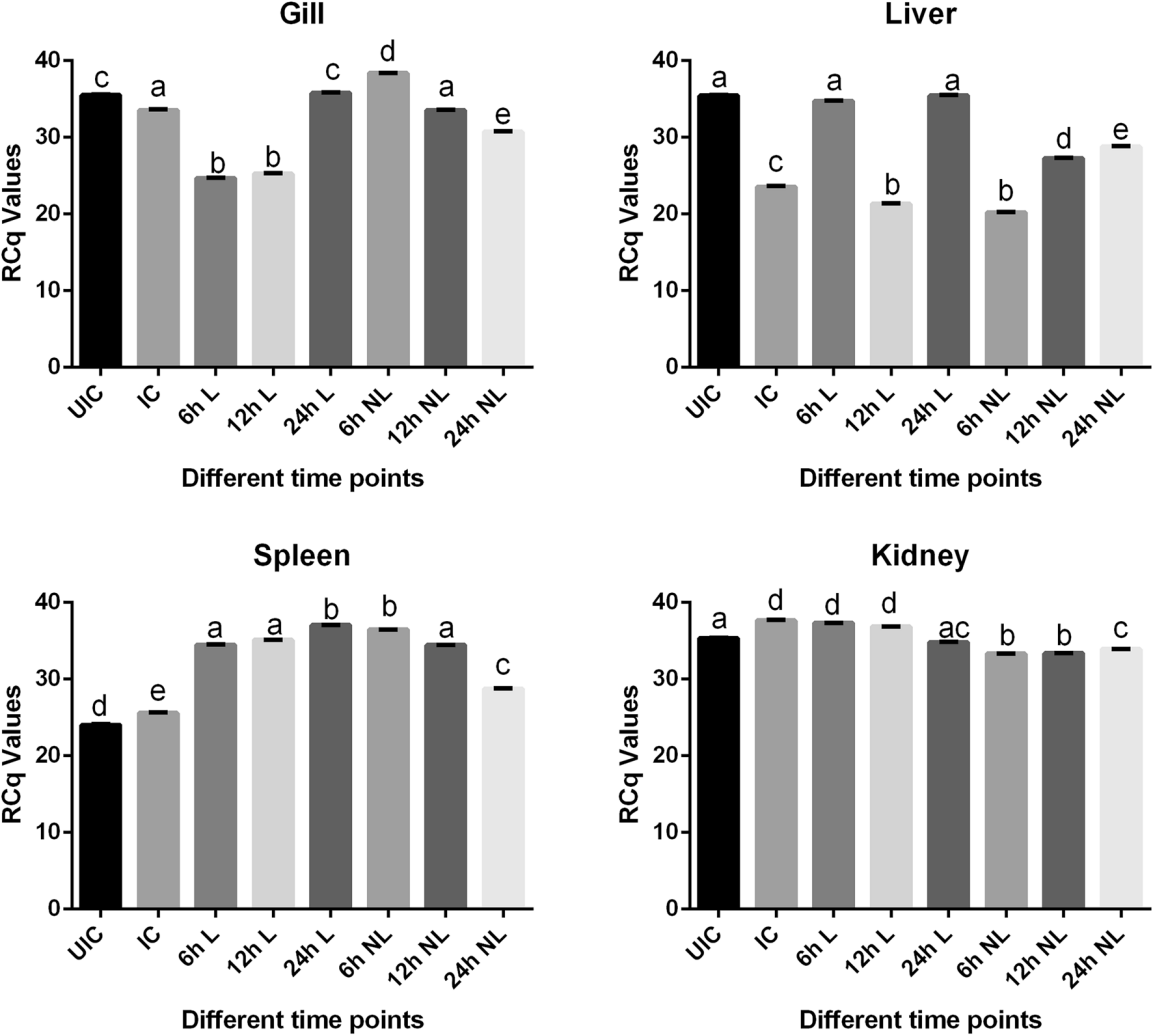
Gene expression analysis of MyD88 in the gills, liver, kidney and spleen of infected fish with *A. hydrophila* and treated with lectin and Pt-lec at different time intervals (6 h, 12 h and 24 h). *UIC* Uninfected control, *IC* infected control, *L* lectin treated, *NL* lectin-PtNPs hybrid treated.

Myd 88 gene expression (Fig. 6) in the case of IC was found to be lower than UIC in gills (− 1.05-fold) and liver (− 1.5-fold), whereas in spleen and kidney the expression was slightly higher. This again shows a tissue-specific expression pattern upon infection. Interestingly, Myd 88 expression was found to be enhanced in all the tissues, except kidney, when infected fish were treated with either L or Pt-lec for different time points. In the case of gills, treatment with L alone led to an increase by 1.06-fold after 24 h of treatment, whereas for Pt-lec, after 6 h, there was a 1.14-fold increase in gene expression. For both liver and spleen too, L and Pt-lec treatment produced good enhancement in Myd 88 expression with Pt-lec producing a slower increase in expression in the case of liver, when compared to L treatment for the same. In the case of spleen, Myd 88 expression was found to be higher after 24 h of treatment with L (1.44-fold), while with Pt-lec levels were found to be highest within 6 h of treatment (1.42-fold), after which it decreased. Thus, in general Pt-lec treatment appeared to be better than L treatment alone, in inducing Myd 88 expression much earlier during the course of infection. This could facilitate better immune activation through multiple pathways that Myd 88 coordinates and this need to be explored further.

Cyclooxygenase-2 (COX-2) or prostaglandin-endoperoxide synthase 2 is involved in the conversion of membrane arachidonic acid to prostaglandin H2, which is an important precursor of prostacyclins^25^. Under normal conditions, COX-2 expression is low, but during inflammatory conditions, there is a dramatic increase in COX-2 activity leading to high levels of inflammation, which is essential for effective immune responses^26^. Thus, COX-2 is important for not only pathogen clearance but also for tissue repair and renewal post-infection, all of which are mediated by inflammatory molecules^27^.

**Figure 7:**
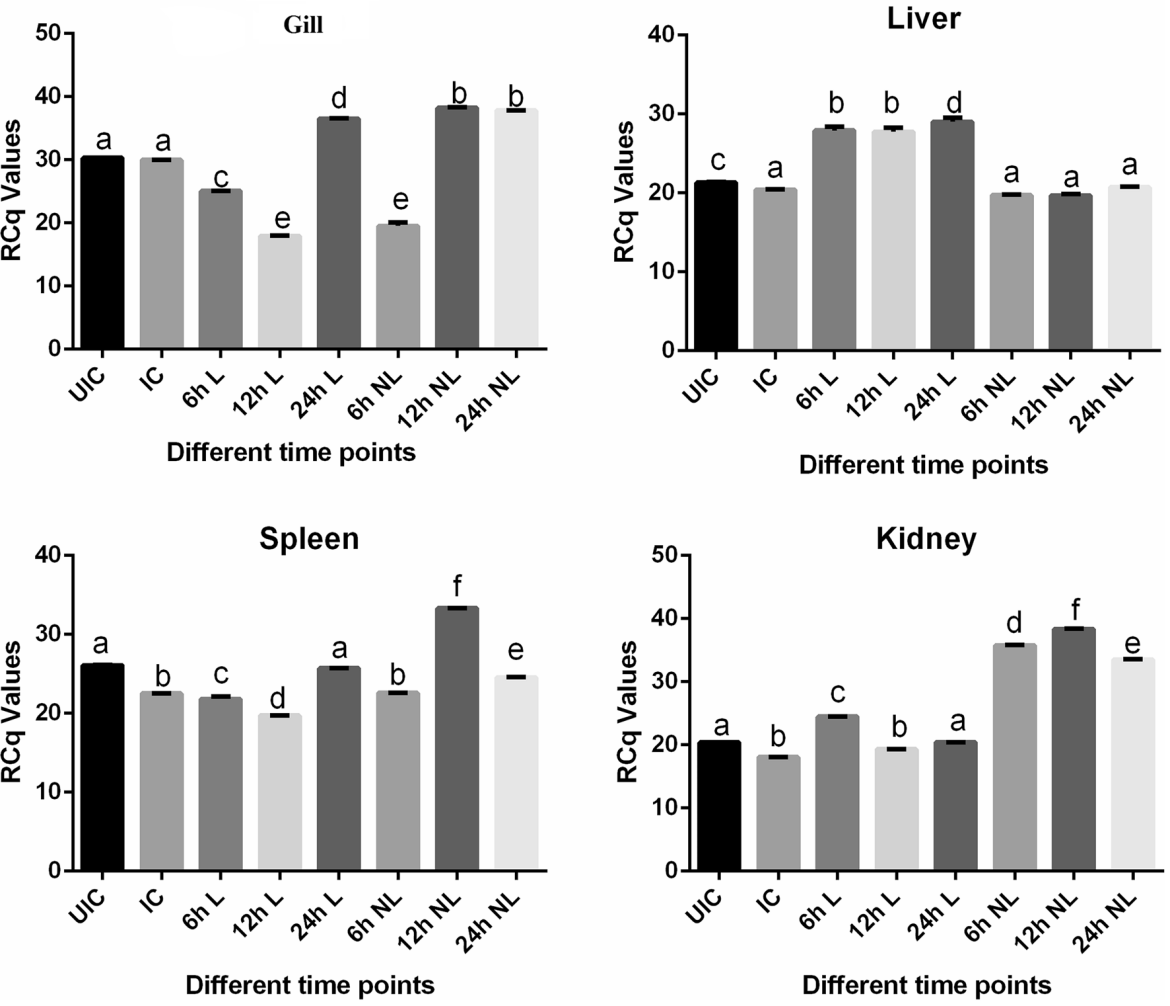
Gene expression analysis of Cox-2 in the gills, liver, kidney and spleen of infected fish with *A. hydrophila* and treated with lectin and Pt-lec at different time intervals (6 h, 12 h and 24 h). *UIC* Uninfected control, *IC* infected control, *L* lectin treated, *NL* lectin-PtNPs hybrid treated.

As far as COX-2 expression in all the tissues is concerned (Fig. 7), it was observed to be lower (avg. − 1.08-fold) in the case of IC when compared to their respective UIC. This suggests that bacterial infection leads to lowering of inflammation, which could be disadvantageous to the host in terms of bacterial clearance. However, when fish were treated with L, there was an increase in the expression of COX-2 in gills (24 h, 1.21-fold), liver (24 h, 1.42-fold), spleen (24 h, 1.14-fold) and kidney (24 h, 1.13-fold). This suggests that lectin treatment had a beneficial effect in terms of triggering COX-2 expression. Interestingly, treatment of fish with Pt-lec appeared to be much more beneficial since it produced better elevation in COX-2 expression at 12 h time point in all the tissues (gills, 1.27-fold; spleen, 1.47-fold; kidney, 2.12-fold), except in liver. Compared to L treatment alone, Pt-lec treatment was more potent in not only stimulating a higher COX-2 expression but this elevation in COX-2 expression was achieved much earlier during the course of infection. This early increase in COX-2 expression could prove to be beneficial in terms of an early onset of inflammatory response, which could help in faster clearance of the pathogen and also facilitate quicker tissue repair. All this could translate into better survival ability for the host. At this juncture, we would like to point out that though the differences observed with Pt-lec treatment were not as dramatic as we believed it could be, the observed increase in gene expression pattern for LEC, Myd 88 and COX-2, could potentially prove to be beneficial in the long term, since Pt-lec treatment was able to stimulate three different immune genes, all of which are critical for catalyzing different mechanisms underlying host response to invading pathogens. This is in addition to our results on the better ability of Pt-lec treatment to reduce in vivo bacterial load and bacteria-induced mortality. Taken together, our results clearly suggest the usefulness of Pt-lec conjugates as potential immunostimulants for aquaculture animals.

## Conclusion

In this study we demonstrated that the MIC of PtNPs could lowered by twofold through forming non-covalent complex with Md-Lec. As prepared Pt-lec hybrid works effectively against fish specific pathogens. In vitro mechanistic study reveals that Pt-lec affects the bacterial membrane integrity and also produces excess ROS to exert the antimicrobial activity. The hybrid was effective in treating bacteria infected Nile Tilapia through reducing the bacterial bioburden in the tissues. Immunological study reveals that Pt-lec attenuates immune related genes in the infected fish to combat bacterial infection. The results spreads light to the possibilities explore lectin and NPs hybrid as promising therapeutic agents after further proper investigations.

## Material and methods

All experiments were performed in compliance with the guidelines as prescribed by the Institutional Animal Ethics Committee (SOST/PhD001/2019) of Kerala University of Fisheries and Ocean Studies, India. The experiments were carried out in compliance with Central Act 26 of 1982 guidelines approved by institute ethical committee. The bacterial isolates used in this study were isolated and identified from diseased fishes previously and they are cultured by incubating at 37 °C for 24 h.

### Preparation of Md-Lec and PtNPs hybrid

Md-*Lec* was isolated from the haemolymph of the Kadal shrimp, *Metapenaeus dobsoni* and purified by mannose-sepharose CL 4B affinity chromatography as described by Rubeena and Preetham^10^. The purity and molecular mass was judged by SDS-PAGE. Platinum nanoparticles were prepared through reducing hexachloroplatinic acid hexahydrate. Briefly, 1 mM hexachloroplatinic acid hexahydrate was dissolved in aqueous solution of pectin (1 mg/mL). The addition of 10 mM sodium borohydride to this mixture turns the solution color to brownish black in 6 h. The formation of nanodimension platinum was judged by transmission electron microscopy. As prepared PtNPs was stored at room temperature and used in the subsequent study. The non-covalent complex formation between Md-Lec and PtNPs was evaluated by fluorescence quenching method. Typically, 3 mL of Md-Lec was titrated with small aliquots of PtNPs (1 mM). The change in fluorescence emission at 342 nm was analyzed by Stern–Volmer plot. The excitation wavelength was 280 nm and emission spectra were recorded in JASCO spectrofluorometer FP-8200.

### Rezasurin microtitre assay (REMA)

The minimum inhibitory concentration (MIC) of PtNPs and Pt-lec hybrid against three aquatic pathogens *A. hydrophila, E. fecalis*, and *S. iniae* were determined by REMA method^11^. Briefly, 100 μL PtNPs (100 μM) was serially diluted in 96-well plate and adjusted the final volume to 100 μL. The wells were seeded with 100 μL of 1 × 10^5^ CFU/mL bacterial cells and cultured for 24 h at 37 °C. Then, 30 μL resazurin solutions (0.01% w/v) was added to each well and cultured further for 2 h at 37 °C. The wells with viable cells showed color change from blue to pink, which is marked as MIC. About 25 μM Md-lec was used for preparing Pt-lec hybrid.

### Membrane permeability assay

The outer membrane (OM) permeability of Gram-negative pathogens was determined by fluorescence probe uptake assay using 1-*N*-phenylnapthylamine (NPN), whereas, the membrane integrity of Gram-positive pathogens was judged using propidium iodide (PI)^13^. Briefly, 1 × 10^5^ CFU/mL bacterial cells were treated with 1× MIC Pt-lec hybrid for 12 h at 37 °C. The cells treated 25 µM Triton X 100 and the untreated cells serves positive control and negative control, respectively. After treatment, the cells were collected, washed twice with phosphate-buffered saline (PBS) buffer and then, treated with 0.5 mM NPN or PI. After 2 h treatment with fluorescence probes, the fluorescence was measured. The excitation wavelength for NPN and PI are 350 and 543 nm, respectively. The emission wavelength for NPN and PI are 420 and 600 nm, respectively.

### Scanning electron microscopy analysis

The bacterial morphological change caused by the treatment with Pt-lec hybrid was assessed by scanning electron microscopy. Typically, the bacteria were cultured in LB media in the absence and presence of Pt-lec hybrid. After 12 h, the culture was fixed by 2.5% gluteraldehyde for 1 h, followed by washing with PBS buffer. Then, the fixed bacteria were dehydrated with ethanol and air dried. The bacterial image was recorded in TESCAN SEM.

#### Lipid peroxidation assay

The lipid peroxidation product like malondialdehyde (MDA) formed in bacterial membranes due to excess generation of reactive oxygen species was determined by Thiobarbituric acid (TBA) assay^10^. About 1 mL of the 1 × MIC PtNPs and 1 × MIC Pt-lec hybrid for 2 h and then, treated with 10% trichloroacetic acid. To this cells, 0.67% TBA was added and incubated for 1 h at 95 °C. After cooling down the reaction mixture to room temperature, the cells were centrifuged at 6000 rpm for 15 min. The reaction mixture was cooled down to room temperature and then centrifuged at 6000 rpm for 15 min. The absorbance of the supernatant was recorded at 532 nm. Untreated cells and cells treated with 25 µM hydrogen peroxide were served as negative and positive control, respectively.

### In vivo antibacterial studies

Optimizations of bacterial dosage for establishment of infection were done previously^11^. Infection was done intramuscularly and treatment was given from the opposite side to that of the infection^13^. The in vivo bacterial infection and the treatment with Pt-lec hybrid was performed in Nile Tilapia that were divided into 3 groups; Group A: Bacterial infected, Group B: Bacterial infected + treated with Pt-lec hybrid, Group C: Control, uninfected fish. Each group consisted of 20 fish. In a typical experiment, 10 μL of 0.1 OD culture of *A. hydrophila* was used for infection. The fish were fed normally and allowed to rest for a period of 3 h. After 3 h of infection with bacteria, Group B was injected with 10 μL of Pt-lec. The fish were monitored for mortality due to bacterial infection for a period of 24 h. At regular time points, (3 h, 6 h, 12 h) one fish from each group was removed, anesthetized and sacrificed. Approximately 100 mg of muscle tissue was dissected and homogenized in sterile PBS. The homogenate was serially diluted using sterile PBS and plated on sterile LB agar plates. The plates were incubated for 24 h at 37 °C. After 24 h, bacterial colonies were counted and reported. All the experiments were performed in triplicates.

### Immune gene expression analysis

The fish were fed normally and after 3 h of infection with bacteria, 10 μL of samples were injected as described above. At regular time points, (6 h, 12 h and 24 h) random fishes from each group was removed, anesthetized and sacrificed. Gills, liver, kidney and spleen were dissected and homogenized in sterile PBS. The total RNA was isolated by Trizol method and then the cDNA was synthesized using Bio-Rad cDNA synthesis kit following the manufacturer’s instructions. The cDNA samples were used to perform reverse transcriptase PCR to study the expression of the selected immune genes (COX2, MyD88 and LEC). The house keeping gene used was EF-1 α. The details of the primers designed for the present study is given in the Table 1 and was purchased from Sigma-Aldrich, India. The PCR products were then loaded onto 1.5% agarose gel.

**Table 1:** Primers used for RT-PCR.

Gene name	Primer name	Accession number	Primer sequence	Amplification
MyD88	MyD88 283 F MyD88 283 R	KT206230.1	GCTGGAACAGACGGAGTA ATCGCAAATGGTGAGAAA	283 bp
COX2	COX2 205 F COX2 205 R	NC_029832.1	TTCTCATTGCCCTTCCTT ATGCGATGGTCTGTTTCT	205 bp
LEC	LEC 135 F LEC 135 R	XM_019351728.2	CCTACACCGCTGTCCTTC GCTCTTCTTACCACCCTTG	135 bp

### Ethical approval

All experiments were approved by the Institutional Animal Ethics Committee (SOST/PhD001/2019) of Kerala University of Fisheries and Ocean Studies, India. The experiments were carried out in compliance with Central Act 26 of 1982 guidelines approved by institute ethical committee and Use Guidelines and the Animal Research of in vivo Experiments (ARRIVE) guidelines.

## Supplementary Information

Supplementary Information.

## Data Availability

All data generated or analysed during this study are included in the article and in the supplementary information. The raw datasets used and/or analysed during the current study are available from the corresponding author upon reasonable request.
