# Supplementary material for: Hybrid of Metapenaeus dobsoni lectin and platinum nanoparticles exert antimicrobial and immunostimulatory effects to reduce bacterial bioburden in infected Nile tilapia
Source: Sci Rep. 2023 Jan 11;13:525. doi: 10.1038/s41598-022-26719-5 (PMC9834305; doi:10.1038/s41598-022-26719-5)
Supplement: Supplementary file 1 — Supplementary Information. [file 41598_2022_26719_MOESM1_ESM.doc]

| Gills |
| --- |
| 1 2 3 4 5 6 7 8 9 |
| 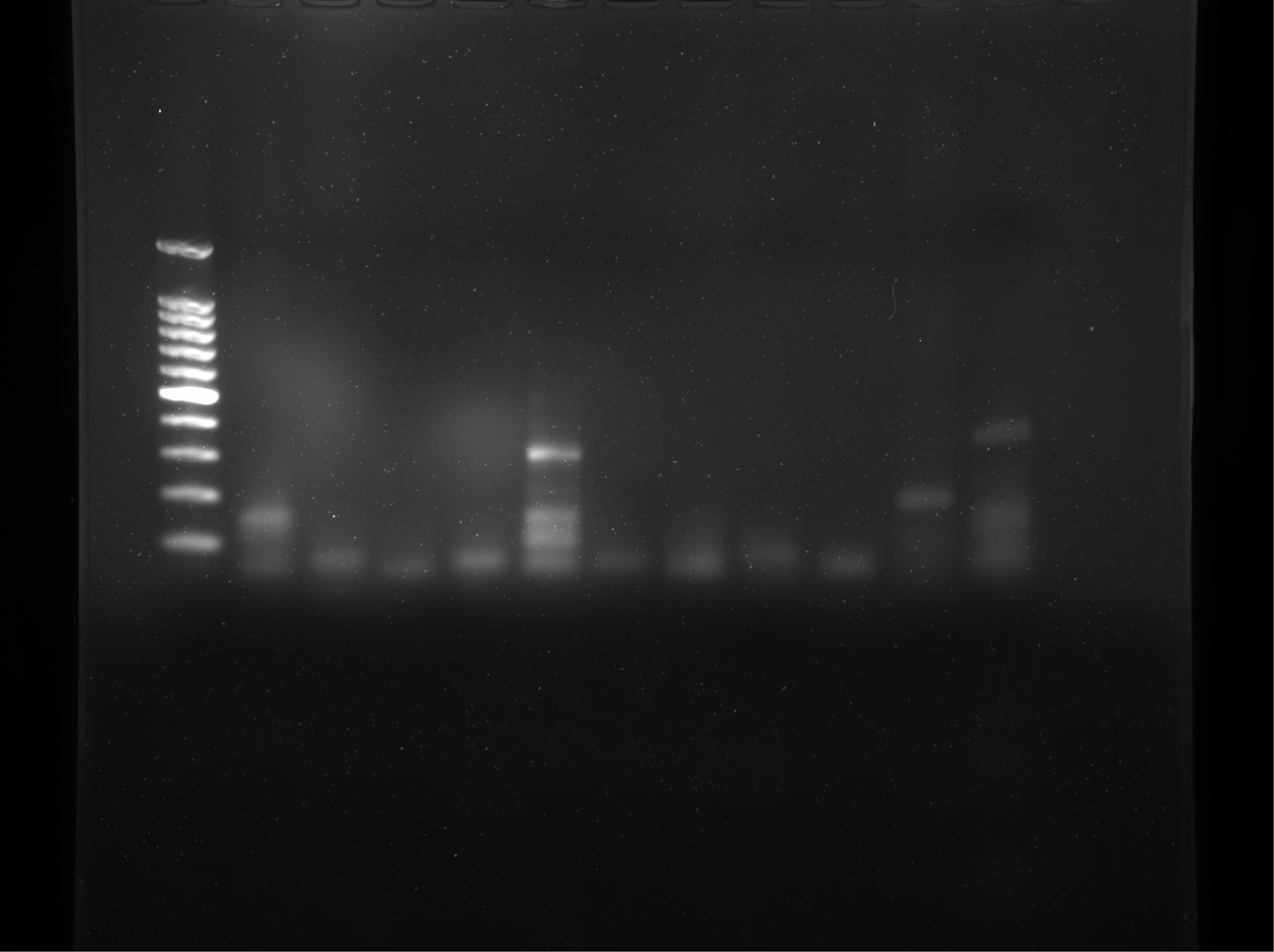 COX2 |
| 1 2 3 4 5 6 7 8 9 |
| 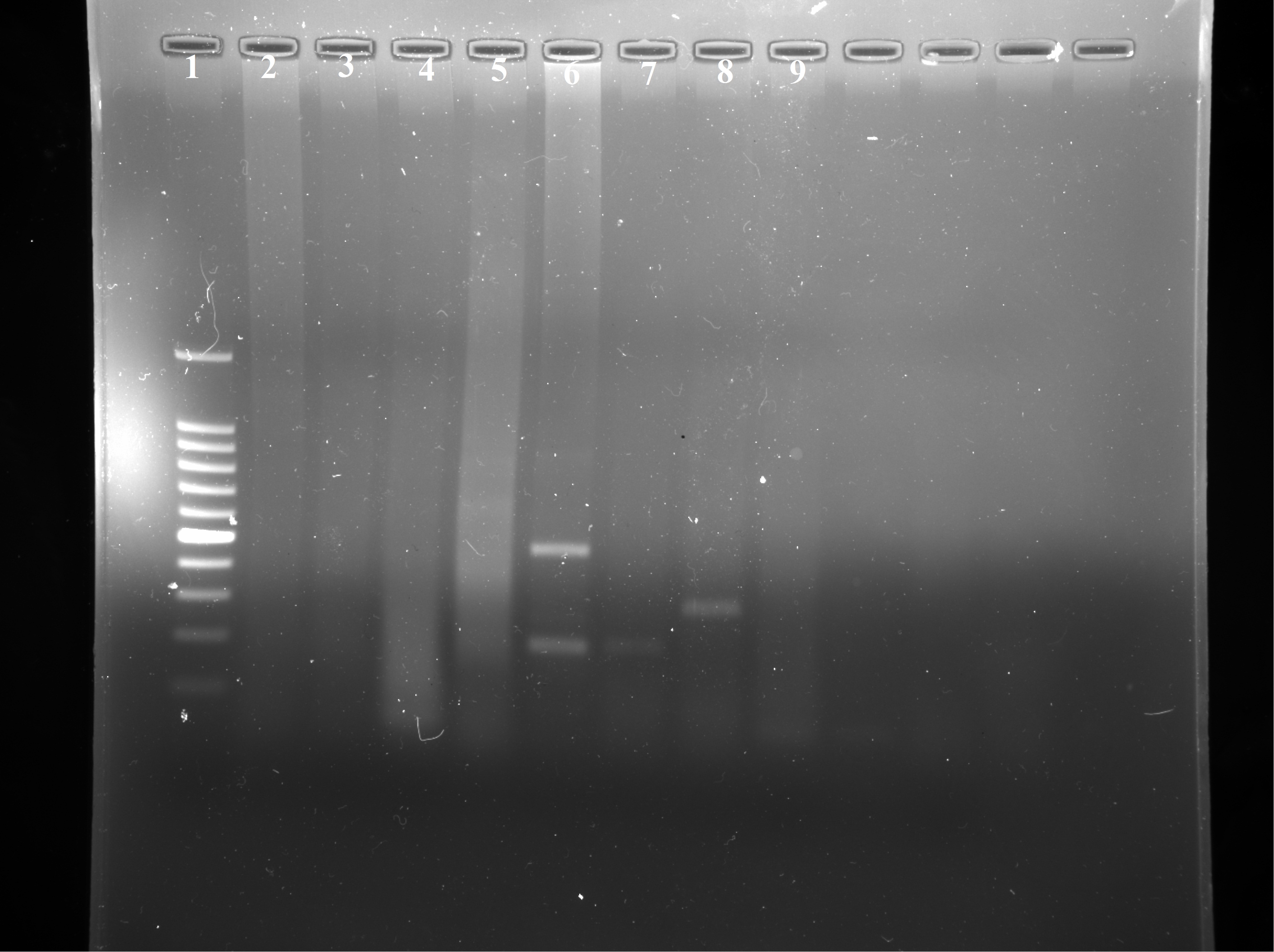 MyD88 |
| 1 2 3 4 5 6 7 8 9 |
| . 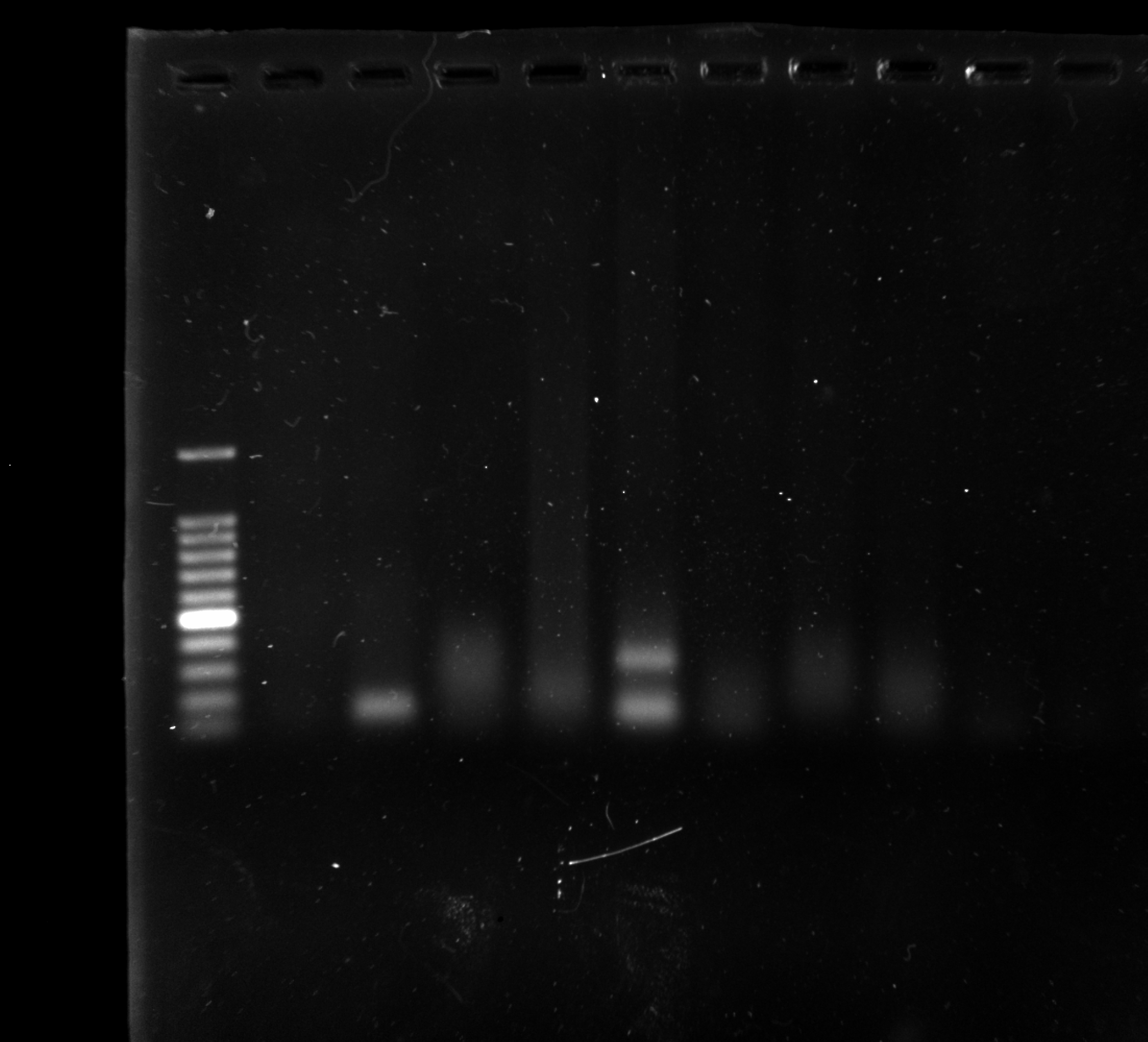 LEC |

Fig. E1: Gene expression analysis in the gills of infected fish with *A. hydrophila* and treated with lectin and Pt-lec at different time intervals. 1 –ladder, 2 – 6h lec, 3 – 12 lec, 4 – 24 lect, 5 – 6h Pt-lec, 6 – 12 Pt-lec, 7 – 24 Pt-lect, 8- uninfected control, 9 – infected control

| Liver |
| --- |
| 1 2 3 4 5 6 7 8 9 |
| 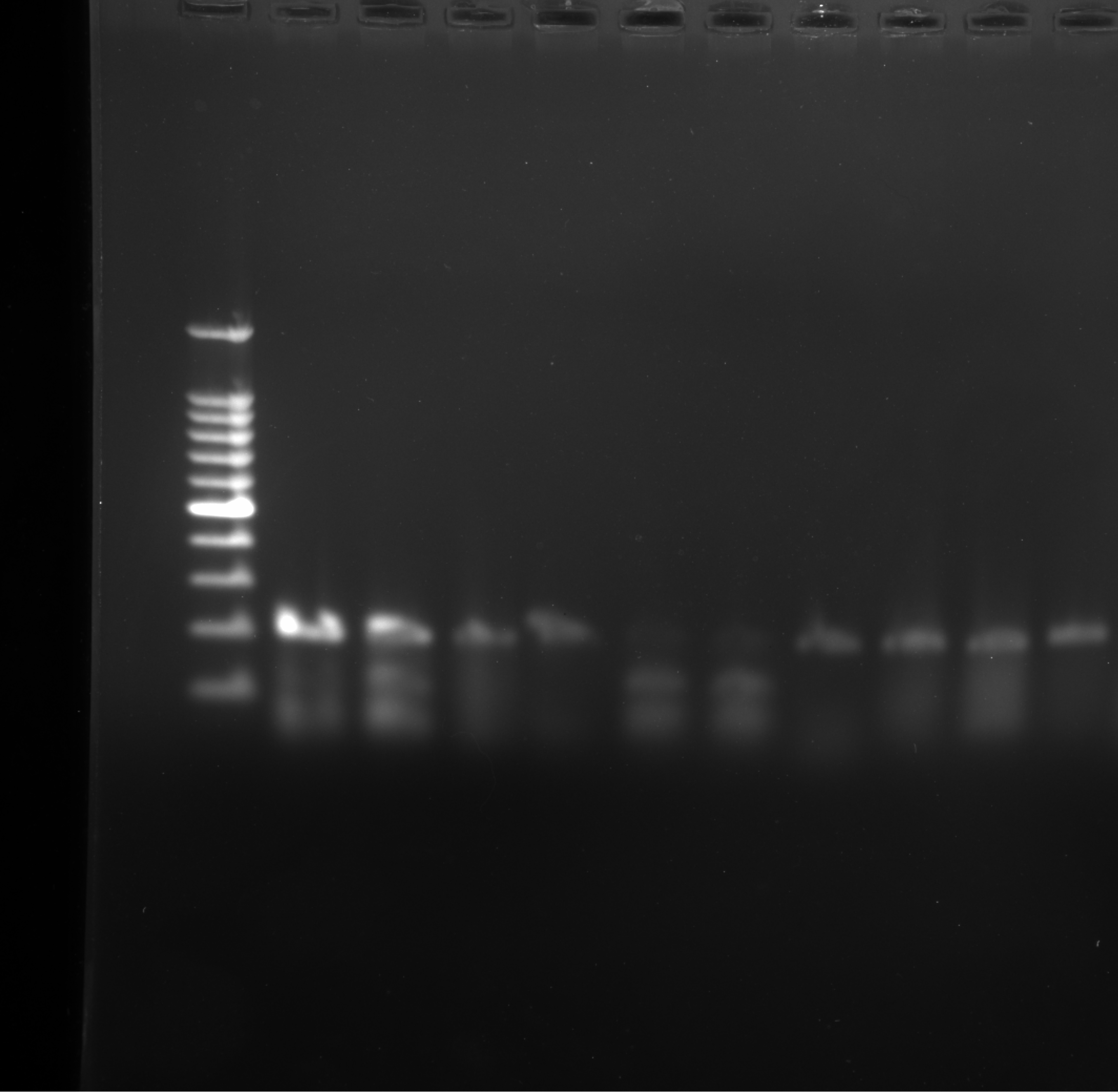 COX2 |
| 1 2 3 4 5 6 7 8 9 |
| 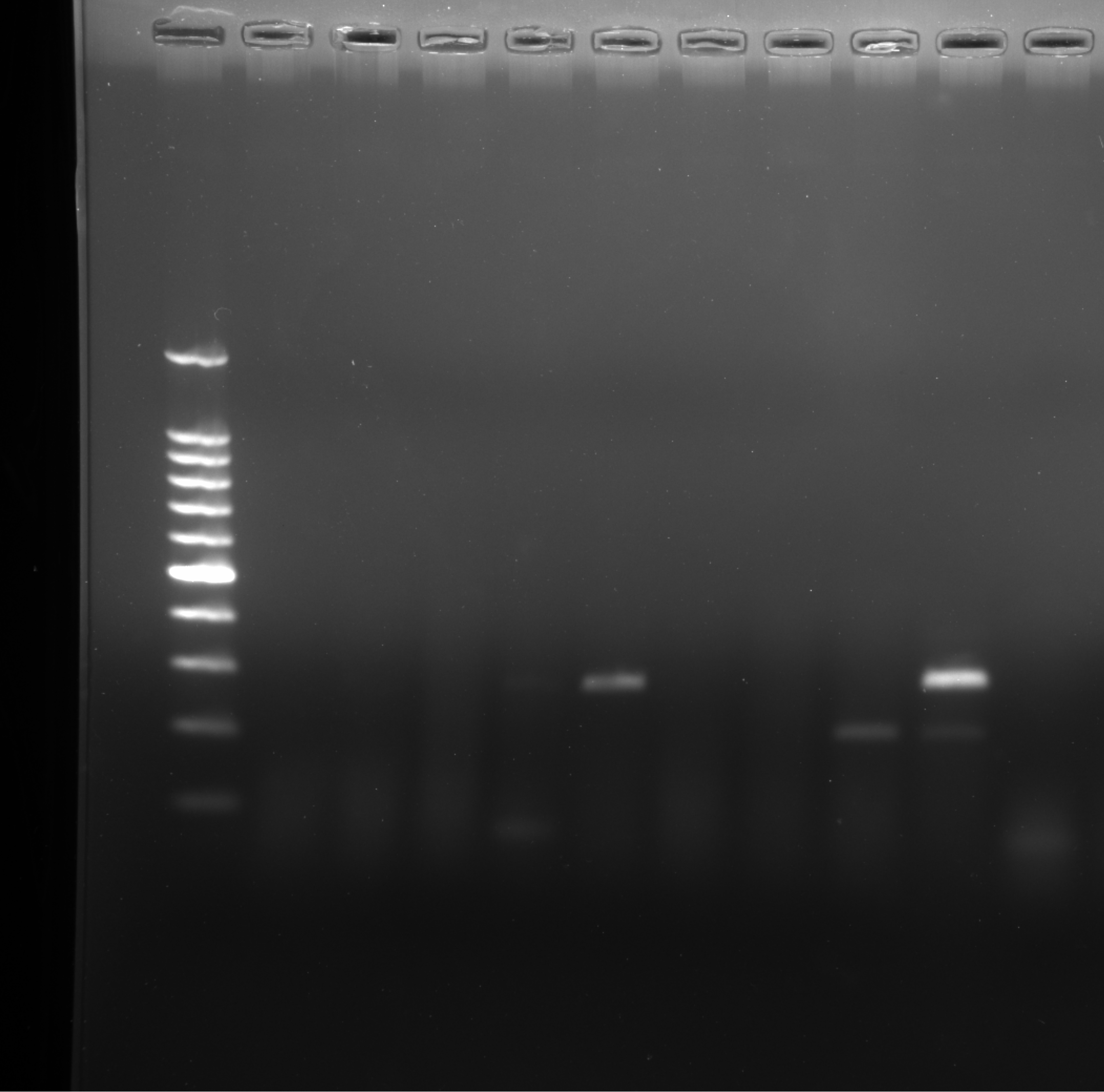 MyD88 |
| 1 2 3 4 5 6 7 8 9 |
| . 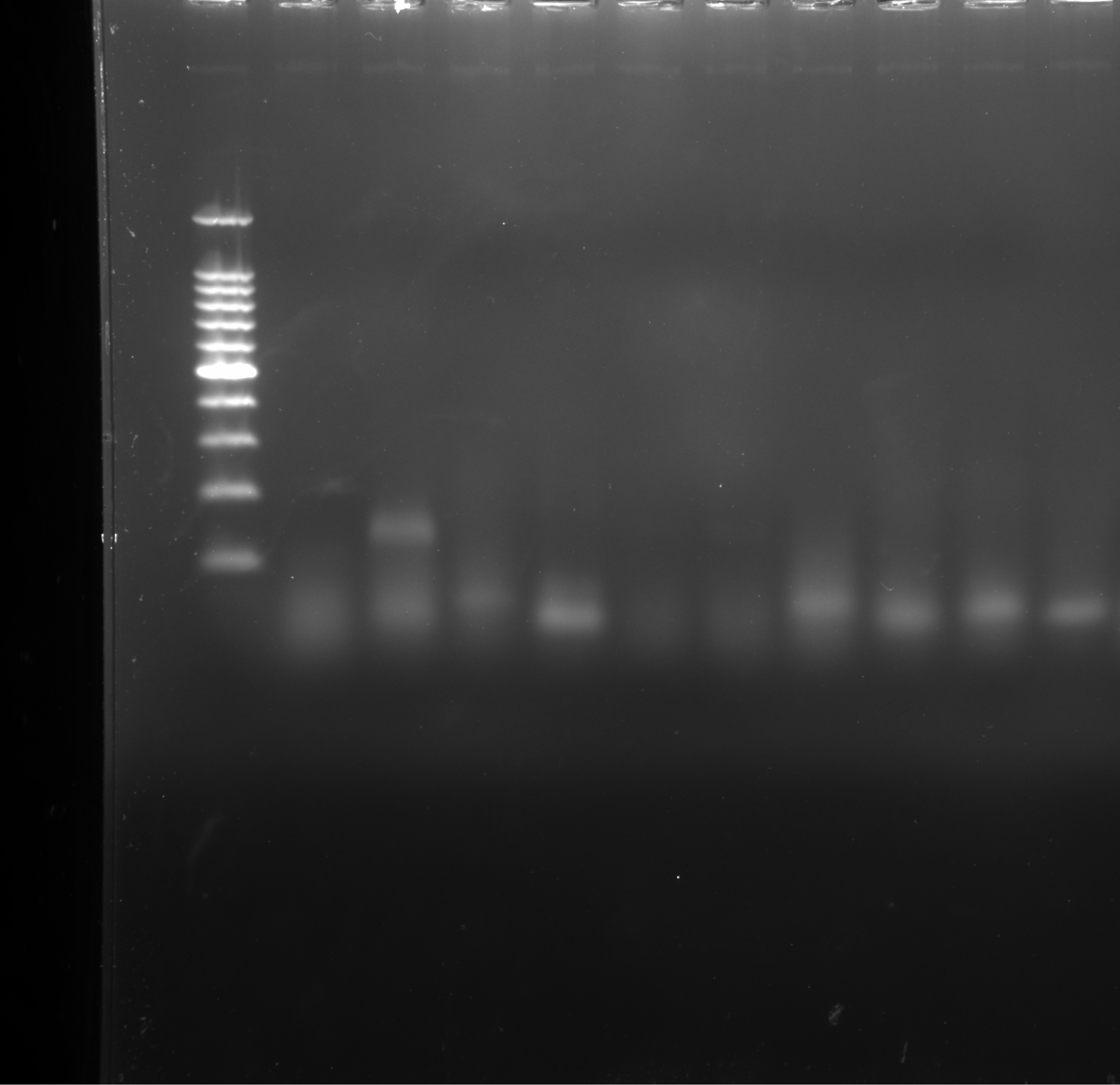 LEC |

Fig. E2: Gene expression analysis in the liver of infected fish with *A. hydrophila* and treated with lectin and Pt-lec at different time intervals. 1 –ladder, 2 – 6h lec, 3 – 12 lec, 4 – 24 lect, 5 – 6h Pt-lec, 6 – 12 Pt-lec, 7 – 24 Pt-lect, 8- uninfected control, 9 – infected control

| Kidney |
| --- |
| 1 2 3 4 5 6 7 8 9 |
| 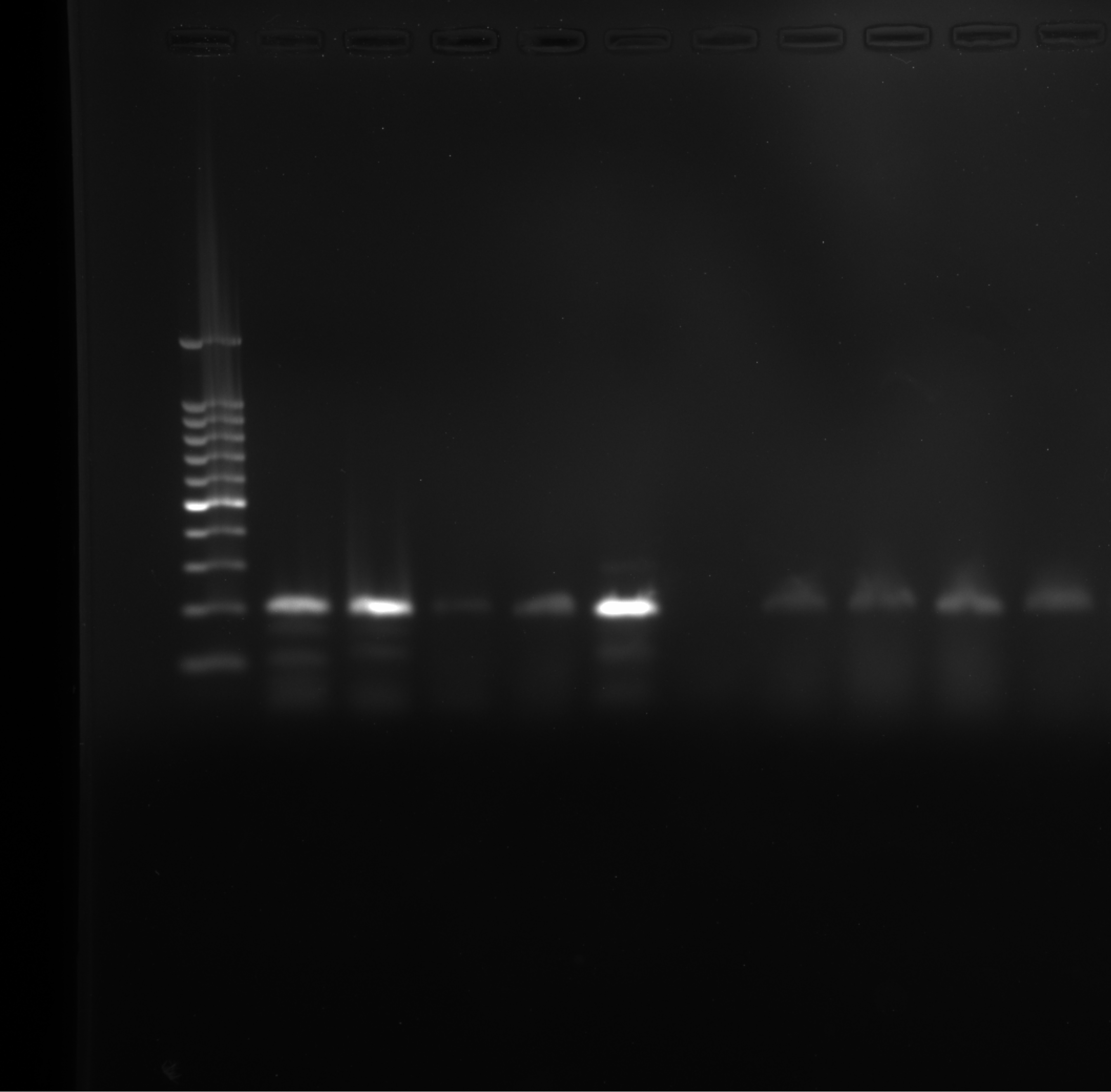 COX2 |
| 1 2 3 4 5 6 7 8 9 |
| 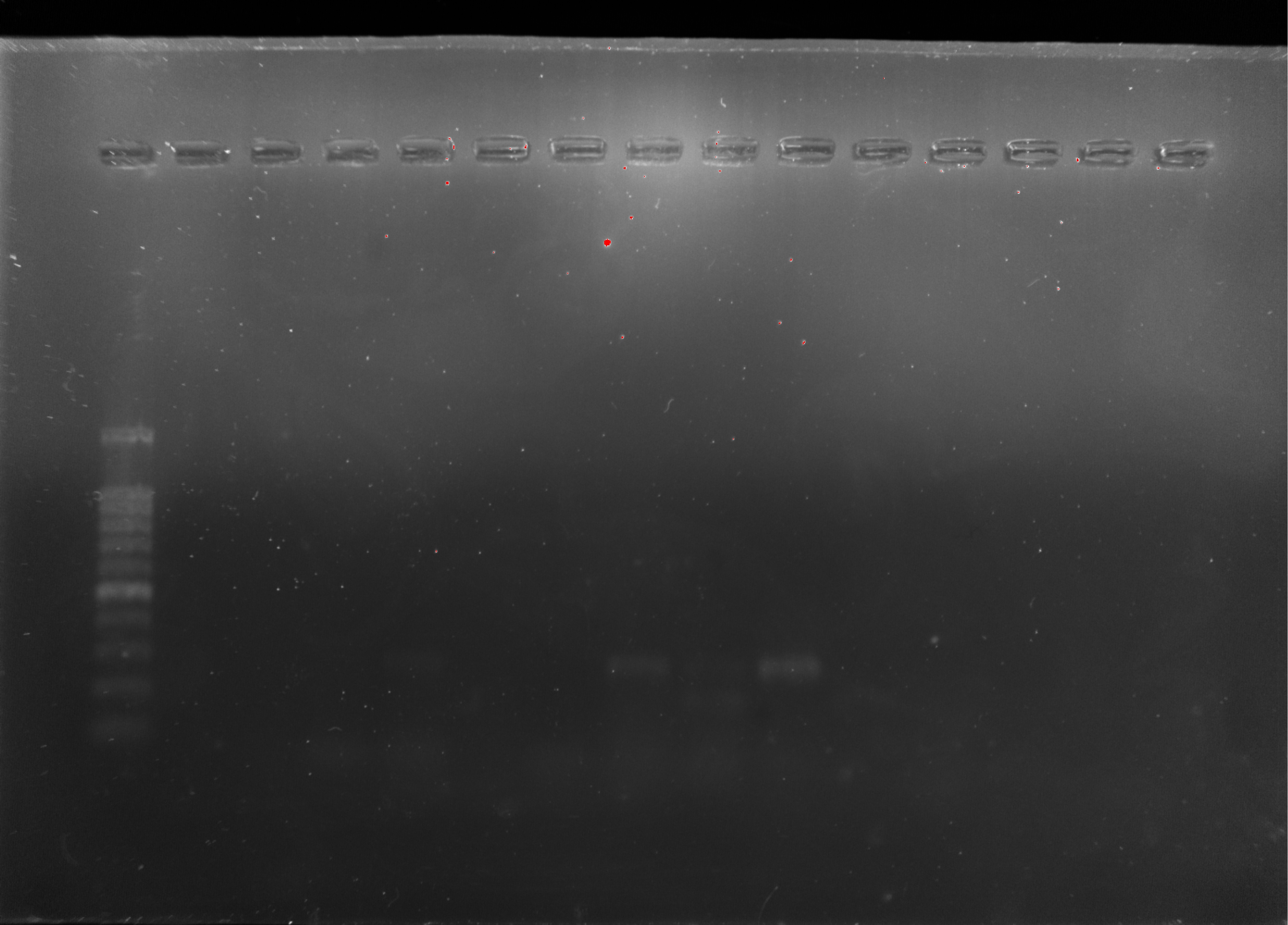 MyD88 |
| 1 2 3 4 5 6 7 8 9 |
| . 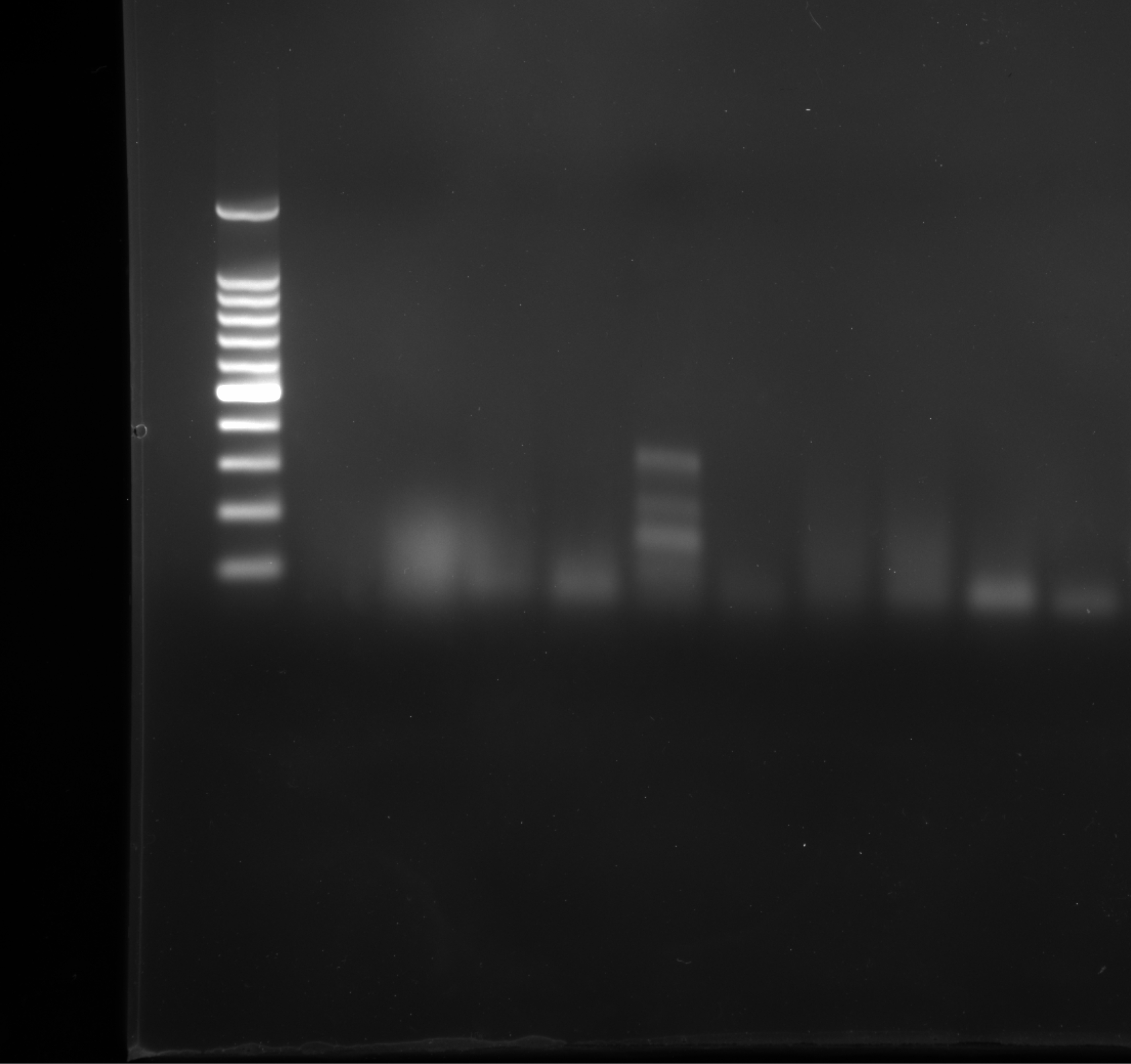 LEC |

Fig. E3: Gene expression analysis in the kidney of infected fish with *A. hydrophila* and treated with lectin and Pt-lec at different time intervals. 1 –ladder, 2 – 6h lec, 3 – 12 lec, 4 – 24 lect, 5 – 6h Pt-lec, 6 – 12 Pt-lec, 7 – 24 Pt-lect, 8- uninfected control, 9 – infected control
